# ﻿Taxonomic and molecular characterization of *Pseudosteringophorusprofundis* sp. nov. (Digenea, Fellodistomidae), a parasite of *Macrourusholotrachys* Günther, 1878 (Gadiformes, Macrouridae) from the deep sea southeastern Pacific Ocean

**DOI:** 10.3897/zookeys.1221.135086

**Published:** 2024-12-31

**Authors:** Marcelo E. Oliva, Fabiola A. Sepúlveda, Rubén Escribano, Luis A. Ñacari

**Affiliations:** 1 Instituto Ciencias Naturales Alexander von Humboldt, Universidad de Antofagasta, Angamos 601, Antofagasta, Chile Universidad de Antofagasta Antofagasta Chile; 2 Instituto Milenio de Oceanografía, Universidad de Concepción, Concepción, Chile Universidad de Concepción Concepción Chile; 3 Laboratorio de Eco – Parasitología y Epidemiología Marina, Facultad de Ciencias del Mar y Recursos Biológicos, Universidad de Antofagasta, 601 Angamos, Antofagasta 1240000, Chile Universidad de Antofagasta Antofagasta Chile; 4 Laboratorio de Ecología y Evolución de Parásitos, Facultad de Ciencias del Mar y Recursos Biológicos, Universidad de Antofagasta, 601 Angamos, Antofagasta 1240000, Chile Universidad de Concepción Concepción Chile

**Keywords:** 28S rDNA, Bigeye grenadier, cox1 mDNA, deep-sea fishes, gallbladder parasite, integrative taxonomy, new species, southeastern Pacific Ocean

## Abstract

*Pseudosteringophorusprofundis***sp. nov.** a new species of deep-sea digenean, parasitizing the gallbladder of the “Bigeye grenadier” (*Macrourusholotrachys* Günther, 1878) in the deep waters of the southeastern Pacific Ocean is described on the basis of morphological and molecular (28S rRNA) data. The new species is distinguishable from *Pseudosteringophorushoplognathi* Yamaguti, 1940, the only other member of the genus, by its subterminal oral sucker, the position of the ovary and testes, the larger anterior seminal vesicle compared to the posterior one, and its larger eggs. In addition, the new species is a parasite of a deep-sea fish, whereas *P.hoplognathi* is a parasite of shallow-water fish. A phylogenetic tree, based on 28S rDNA sequences, indicates that this species is included in a clade of deep-sea fellodistomid species (*Steringophorus* spp.). We provide the first molecular data on the genus *Pseudosteringophorus* Yamaguti, 1940 and expand the molecular database for the family Fellodistomidae. Further studies, including sequences from other fellodistomid taxa, are needed to more precisely infer relationships within this family.

## ﻿Introduction

The deep sea is one of the world’s most vulnerable and unexplored ecosystems and is considered an important reservoir of biodiversity (Danovaro et al. 2010; Ramirez-Llodra et al. 2010). Knowledge of this biodiversity remains scarce (Danovaro et al. 2010) and this is particularly true for the deep waters of the southeastern Pacific Ocean (SEPO) (Danovaro et al. 2002; Sabbatini et al. 2002; Gambi et al. 2003; Fujii et al. 2013; Weston et al. 2021; Ramírez-Flandes et al. 2022). Parasites are a critical component in aquatic ecosystems and play important roles in the food web and the population dynamics of hosts (McLaughlin et al. 2020). Knowledge of metazoan parasites, especially Digenea in deep-water fishes from SEPO, is limited (Rodriguez and George-Nascimento 1996; Oliva et al. 2008; Salinas et al. 2008; Ñacari and Oliva 2016; Espínola-Novelo et al. 2018; Ñacari et al. 2022).

A few families of digeneans (Fellodistomidae Nicoll, 1909, Gonocercidae Skrjabin & Guschanskaja, 1955, Gorgoderidae Looss, 1899, Hemiuridae Looss, 1899, Lecithasteridae Odhner, 1905, Lepidapedidae Yamaguti, 1958, Opecoelidae Ozaki, 1925, Zoogonidae Odhner, 1902) are reported from the deep sea, especially in bathyal areas (>1000 m) (Bray 2020). So far, 23 species of digeneans are recorded as parasites of fishes of the genus *Macrourus* (Gadiformes) of which nine species parasitize *Macrourusholotrachys* Günther, 1878 (Münster et al. 2016; Ñacari et al. 2022). In this study, we describe a new species of digenean, *Pseudosteringophorusprofundis* sp. nov. (Fellodistomidae) from the gallbladder of *M.holotrachys* collected in the deep sea off northern Chile based on morphological and molecular analyses.

## ﻿Material and methods

### ﻿Collection and morphological analysis

Thirty-six adult specimens of *M.holotrachys* were obtained periodically during 2017 as bycatch from the artisanal longline fishery (9.26 km length) of the Patagonian toothfish, *Dissostichuseleginoides* Smitt, 1898, in northern Chile (≈ 22°30'S, 70°40'W) at depths between 1000 and 2000 m. The fish were frozen onboard at −18 °C immediately after capture and transported to the parasitology laboratory at the Universidad de Antofagasta for further analysis. Digeneans were removed from the gallbladder, fixed in AFA (ethanol: formalin: acetic acid), preserved in 70% ethanol and stained with acetocarmin or Gomori’s thrichrome, dehydrated in an alcohol series (70% to 100%), cleared in oil of clove® (Sigma–Aldrich, Madagascar) and mounted in Entellan (Merck-Millipore, Billerica, Massachusetts). Illustrations were prepared with Adobe Illustrator CS9 from draft line drawings made with a camera lucida. Measurements are in micrometres and are given as the range followed by the mean in parentheses. Taxonomic identification of fellodistomids follows Bray (2002). Paratypes of *Pseudosteringophorushoplognathi* and *Benthotremahoplognathi* Yamaguti, 1938 (MPM coll. 23037 and coll. 230370, respectively) were examined.

### ﻿DNA extraction, amplification and sequencing

DNA was isolated from two Fellodistominae specimens following a modified version of the salting out protocol (Miller et al. 1988). This involved treatment with sodium dodecyl sulphate, digestion with proteinase K, NaCl protein precipitation, and subsequent ethanol precipitation. The DNA was eluted in nuclease-free water and quantified using a BioSpec-nano spectrophotometer (Shimadzu, Japan).

For the molecular analyses, regions within the 28S ribosomal DNA large subunit (LSU rDNA) and the mitochondrial cytochrome *c* oxidase 1 gene (*cox1* mDNA) were amplified by polymerase chain reaction (PCR). The LSU rDNA region 28S was amplified by PCR using the forward primer C1 (5′-ACCCGCTGAATTTAAGCAT-3′) and reverse primer D2 (5′-TGGTCCGTGTTTCAAGAC-3′) (Chisholm et al. 2001); *cox1* mtDNA was amplified using the forward primer JB3 (5’- TTTTTTGGGCATCCTGAGGTTTAT-3’) and the reverse primer COX1 (5’-AATCATGATGCAAAAGGTA-3’) (Leung et al. 2009). The reaction was carried out in a final volume of 35 µL comprising five standard units of GoTaq DNA polymerase (Promega), 7 µL of 5× PCR buffer, 5.6 µL of MgCl_2_ (25 mM), 2.1 µL of BSA (10 mg/mL), 0.7 µL of deoxynucleotide triphosphate (dNTP; 10 mM), 10 pM of each primer, 3 µL of template DNA, and sufficient nuclease-free H_2_O to make the total volume up to 35 µL. A Boeco Ecogermany M-240R Thermal Cycler (Boeckel, Hamburg, Germany) was used to carry out PCR for LSU rDNA and *cox1* mDNA using the programs reported in Chisholm et al. (2001) and Leung et al. (2009) respectively. The PCR products were sent to Macrogen (Seoul, Korea; http://www.macrogen.com) for purification and sequencing of both the DNA forward and reverse strands. The sequences were edited and contigs were assembled using ProSeq 2.9 beta (Filatov 2002). New sequences obtained were compared with the GenBank databases through a nucleotide BLAST (https://blast.ncbi.nlm.nih.gov/Blast.cgi). All unique sequences obtained during this study were deposited into GenBank (28S rDNA: PQ109082–PQ109083; *cox1* mDNA: PQ110033–PQ110034).

For phylogenetic analysis, new 28S rDNA sequences obtained in this study were aligned with those of 31 members of Fellodistomidae available in GenBank, 28 sequences belonging to Fellodistominae and three sequences belonging to Tergestiinae (Suppl. material 1). The sequence of *Prosogonariumangelae* Cribb & Bray, 1994 (Tandanicolidae Johnston, 1927) was used as an outgroup, following Bray and Waeschenbach (2020). The alignment was performed using Mafft v.7 (Katoh et al. 2019) with the Q-INS-i algorithm. The aligned sequences were then visualized in ProSeq v. 2.91 (Filatov 2002) and trimmed at the ends. Poorly aligned positions were removed using Gblocks 0.91b (Castresana 2000). Phylogenetic reconstruction was performed using Bayesian inference (BI) and maximum-likelihood (ML) analyses. The jModelTest v. 0.1.1 tool (Posada 2008) was used to identify the best evolutionary model under the Corrected Akaike information criterion (Akaike 1973).

The best model for 28S rDNA aligned sequences was GTR+I+G. The BI analyses were conducted using MrBayes v. 3.2.2 (Ronquist et al. 2012) with the following parameters: nst = 6 and rates invgamma according to the evolutionary model determined by jModelTest. The analysis was performed for 10,000,000 generations, with one run of four chains, sampling every 1000 generations. The initial 25% was discarded as burn-in. Visual inspection of log-likelihood scores against generation time was performed in TRACER v. 1.7 (Rambaut et al. 2018). Support for nodes in the BI tree topology was obtained by posterior probability (PP). The ML analyses were performed using W-IQ-TREE (http://iqtree.cibiv.univie.ac.at/ accessed on 18 July 2024), with 1000 bootstrap replicates for statistical support. The trees were visualized and edited in FigTree v. 1.4.4 (http://tree.bio.ed.ac.uk/software/figtree/). Finally, the pairwise p-distances for 28S rDNA were analyzed using the MEGA v. 6 software (Tamura et al. 2013).

Following Muff et al. (2022) we defined the following five categories for BI nodal support as: PP = 1: fully supported; PP = 0.99–0.90: strongly supported; PP = 0.89–0.80: moderately support; PP = 0.79–0.70: weakly supported; PP = < 0.69: not supported.

## ﻿Result

### ﻿Taxonomy

**Family Fellodistomidae Nicoll, 1909**

**Genus *Pseudosteringophorus* Yamaguti, 1940**

#### ﻿ Pseudosteringophorus profundis sp. nov.

Taxon classificationAnimaliaStrigeididaFellodistomidae

A9380EFF-5DE7-562D-8DF2-5461ED8580A2

https://zoobank.org/BCBF8A63-920C-4795-849D-A1F20E7A99B1

Fig. 1A, B

##### Host.

*Macrourusholotrachys* Günther, 1878 (Gadiformes: Macrouridae).

##### Site of infection.

Gallbladder.

##### Type locality.

northern Chile (≈ 22°30'S, 70°40'W), at depth ranging from 1000 to 2000 m.

##### Prevalence.

21 of 36 (39%).

##### Intensity.

1–333 (17).

##### Material examined.

• ***Holotype***: (MPM coll. no. 25292) and two ***paratypes*** (MPM coll. no. 25293) in the Meguro Parasitological Museum, Tokyo, Japan (MPM) • Three ***paratypes*** (MNHNCL PLAT-15073-15075) in the Museo Nacional de Historia Natural, Santiago, Chile • Three ***paratypes*** (MUSM-HEL 5480) in the Museo de Historia Natural, Universidad Nacional Mayor de San Marcos, Lima, Peru (MHN-UNMSM).

##### Representative DNA sequences.

GenBank accession number, 28S rDNA (PQ109082– PQ109083) and *cox1* mDNA (PQ110033–PQ110034).

##### Differential diagnosis.

The new species belongs to the family Fellodistomidae, a large family of marine fish digeneans characterized by restricted fields of vitelline follicles (Bray 2002). The new species *Pseudosteringophorusprofundis* was assigned to the genus *Pseudosteringophorus* based on morphological characteristics typical of the genus, including a fusiform body, intestinal bifurcation in mid-forebody, caeca reaching ventral suckers, internal vesicle seminal bipartite, ovary dextrodorsal and entire, Y-shaped excretory vesicle, and eggs without spines (Bray 2002). Until now, the genus was monotypic, with *Pseudosteringophorushoplognathi* Yamaguti, 1940, from the intestine of *Oplegnathuspunctatus* (Temminck & Schlegel, 1844) from Hamazima, Japan, being the only recorded species. Machida et al. (2007) later examined *P.hoplognathi* from the intestine of *Oplegnathusfasciatus* (Temminck & Schlegel, 1844) at the Tokyo Wholesale Market (NSMT-PI 798), which are larger than Yamaguti´s specimens and divided into two groups (large and small specimens) (Table 1). *Pseudosteringophorusprofundis* sp. nov. differs from both Yamaguti’s and Machida’s specimens of *P.hoplognathi* by: (1) ovary overlapping the right testes and close to the anterior margin of ventral sucker, whereas in *P.hoplognathi* the ovary is pretesticular; (2) testes are asymmetrical, behind and partly overlapping the ventral sucker, while in *P.hoplognathi*, they are symmetrical and situated in the anterior hindbody; and (3) egg size is larger (35–50 × 21–30 µm) compared to *P.hoplognathi* (27–34 × 15–20 µm in Yamaguti (1940), and 21–26 × 13–16 µm in Machida et al. (2007) (Table 1). The site of infection also differs: the intestine for *P.hoplognathi* but gall bladder for the new species. In addition, the new species is a parasite of a deep-sea gadiform whereas *P.hoplognathi* parasitizes a shallow-water centrarchiform.

**Table 1.** Morphometric data comparisons of *Pseudosteringophorushoplognathi* and our specimens of *P.profundis* sp. nov. Measurements are shown in μm with the mean followed by the range (when available).

	*Pseudosteringophorushoplognathi*	*Pseudosteringophorushoplognathi*	*Pseudosteringophorushoplognathi*	*Pseudosteringophorusprofundis* sp. nov.
Definitive host	*Oplegnathuspunctatus*	*Oplegnathusfasciatus*	*Oplegnathusfasciatus*	*Macrourusholotrachys*
Author	Yamaguti 1940	Machida et al. 2007*	Machida et al. 2007*	This study
Specimens examined		10	4	11
Body length	1100–1800	2330–2880	2020–2380	1313–2120 (1747)
Body width	300–480	1150–1430	670–780	673–1030 (873)
Ratio body length:width		2	3.0–3.1	1.7–2.3 (2.0)
Oral sucker length	160–280	340–450	210–240	269–413 (335)
Oral sucker width	100–200	340–420	160–210	299–395 (346)
Pharynx length	39–60			112–211 (147)
Pharynx width	45–54			145–192 (167)
Esophagus length	60–150			32–32 (32)
Ventral sucker length		490–690	260–340	358–561 (456)
Ventral sucker width	175–310	680–900	280–370	377–622 (468)
ratio oral sucker/ ventral sucker	0.65–0.67	1: 1.8–2.4	1:1.6–1.8	1.1–1.8 (1.4)
Forebody length				623–947 (820)
Forebody (%) of body length		48–54	43–52	41.8–53.5 (47.3)
Hindbody length				250–724 (481)
Hindbody (%) of body length				18.0–34.3 (27.0)
Right testes length		280–340	200–290	218–389 (320)
Right testes width		200–280	170–240	129–307 (237)
Left testes length		250–320	210–260	205–432 (319)
Left testes width		180–240	170–210	108–338 (236)
Testes length (average)	110–160	265–330	205–275	220.5–410.5 (319.7)
Testes width (average)	90–140	190–260	170–225	122.5–322.5 (236.5)
Cirrus pouch length	250–360	660–740	520–570	365–617 (522)
Cirrus pouch width	70–135	240–290	180–200	78–169 (135)
Posterior seminal vesicle length	45–60			72–136 (109)
Posterior seminal vesicle width	24–48			58–122 (90)
Anterior seminal vesicle length	50–105			39–79 (52)
Anterior seminal vesicle width	24–60			30–75 (49)
Ovary length	100–150	230–320	180–240	105–197 (155)
Ovary width	70–95	150–200	90–190	82–221 (134)
Eggs length	27–34	21–24	23–26	34.6–49.5 (45.2)
Eggs width	15–20	15–16	13–16	21.4–29.6 (25.8)

* Measurements of larger and smaller specimens of Machida et al. (2007), respectively.

##### Description.

**Figure 1.**
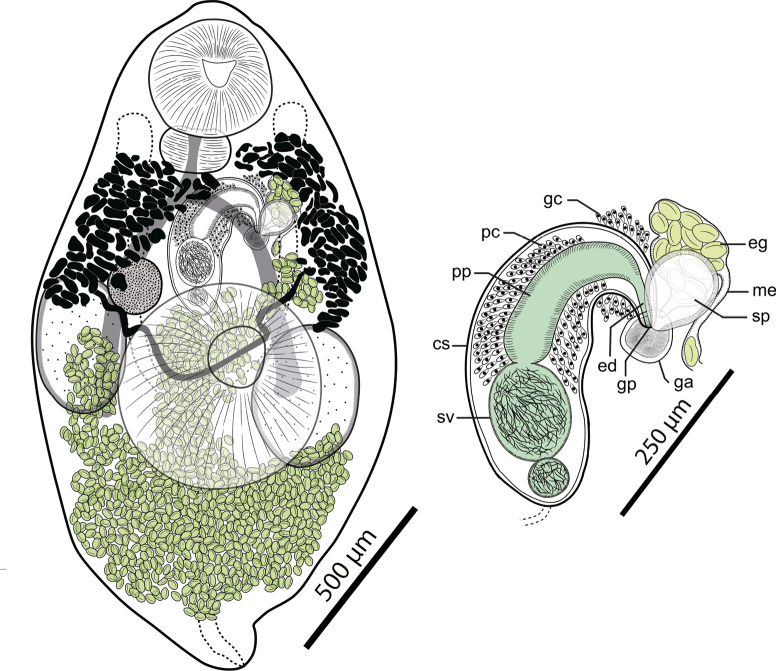
*Pseudosteringophorusprofundis* sp. nov. **A** holotype, ventral view **B** terminal genitalia, ventral view. Abbreviations: gc: glandular cells; pc: prostate cells; pp: pars prostatica; sv: seminal vesicle; ed: ejaculatory duct; gp: genital pore; ga: atrium genital; sp: spermatophore; me: metaterm; eg: eggs.

(Based on 11 stained whole-mounts, Table 1, Fig. 1A, B) Body fusiform, more pointed at posterior than at extremity anterior, 1313–2120 (1747) in length, with maximum breadth of 673–1030 (873) at ventral sucker level. Oral sucker subterminal, rounded, with ventral concavity in lateral view, 269–413 (335) × 299–395 (346). Ventral sucker bowl-shaped, 358–561 (456) × 377–622 (468), near mid-body. Sucker ratio 1.1–1.8 (1.4). Forebody 41.8–53.5 (47.3) % of body length. Prepharynx very short. Pharynx subglobular, 112–211 (147) × 145–192 (167). Esophagus indistinct bifurcates to form intestinal caeca in mid-forebody. Caeca blind, ending at mid-acetabular level.

Testes two, ovoid asymmetrical, one on each body side, posterior to and partly overlapping ventral sucker; right testis 218–389 (320) × 129–307 (237) and left testis 205–432 (319) × 108–338 (236). Cirrus sac elliptical, with anterior end turned sinistral toward genital atrium, with thick wall of inner circular and outer longitudinal muscle fibers, extending to obliquely just inside of right caecum with posterior end passing dorsal to anterior border of ventral sucker, containing seminal vesicle, pars prostatica and short ejaculatory duct opening into genital atrium. Seminal vesicle internal, thin-walled, bipartite, constricted into unequal chambers; anterior chamber 72–136 (109) × 58–122 (90); posterior chamber 39–79 (52) × 30–75 (49). Pars prostatica long, cylindrical surrounded by prostatic cells. Genital pore in left submedian line at anterior part of middle third of body, just ventral and opening to left caecum. Genital atrium wide, muscular. Spermatophore detected attached to genital atrium in several individuals.

Ovary ovoid to spherical, 105–197 (155) × 82–221 (134), dextrodorsal to ventral sucker, near or overlapping right testes. Proximal region of uterus forms uterine seminal receptacle. Mehlis’ gland and Laurer’s canal not observed. Uterus occupies most of post-testicular region, ascends anteriorly between testes, or dorsally to right testis. Metraterm thin-walled, indistinct. Eggs numerous, elongated and oval, operculate, tanned, thick-shelled, 34.6–49.5 (45.2) × 21.4–29.6 (25.8). Vitellarium follicular; follicles numerous, small, closely massed in two fields; fields lie immediately lateral to anterior half of each caecum, between pharynx level and ovarian to anterior border testicular level. Excretory vesicle Y-shaped; branching point obscured by eggs; arms reach just pre-bifurcal.

##### Etymology.

The name “profundis” of the new species refers to the depth at which their hosts were captured.

### ﻿Phylogenetic data

**Figure 2.**
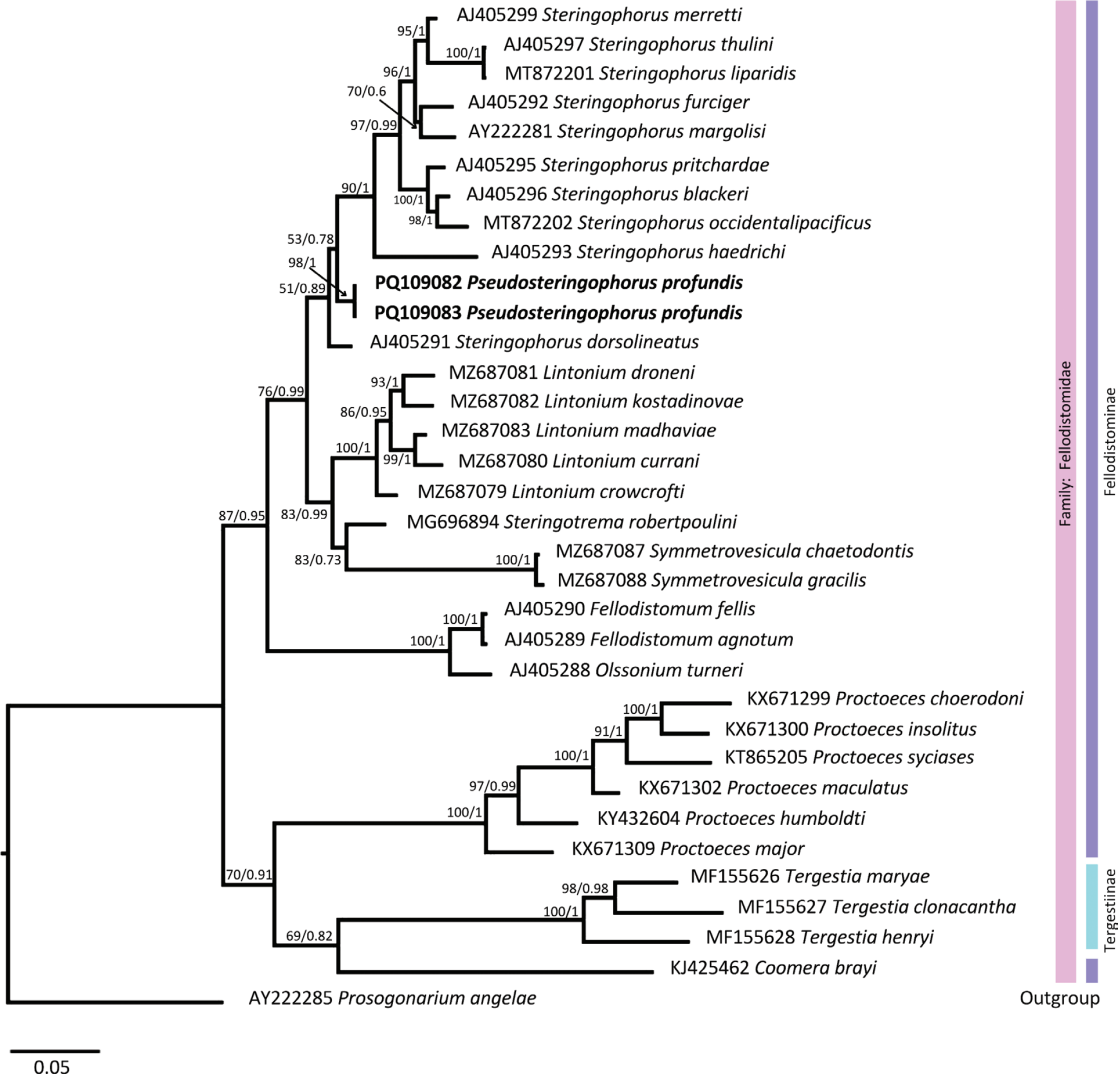
Relationships between fellodistomid taxa based on maximum likelihood (ML) and Bayesian inference (BI) of the partial 28S rDNA dataset. Bootstrap and posterior probability support values are shown at the nodes as ML/BI. The scale bar indicates the expected number of substitutions per site.

Two sequences of 839 base pairs (bp) each were obtained from *Pseudosteringophorusprofundis* sp. nov. for the 28S rDNA gene. No polymorphic sites were detected between the two sequences. The final alignment dataset consisted of 34 sequences of 818 bp in length. Both inference methods, BI and ML, resulted in the same topology but with different statistical support. *Pseudosteringophorusprofundis* sp. nov. was clustered with moderate to weak support (PP = 0.89; ML = 51) within a clade that included ten species of *Steringophorus* (Fig. 2) suggesting that *Steringophorus* is a paraphyletic group. According to genetic distance, the most closely related species to *P.profundis* sp. nov. was *Steringophorusdorsolineatus* (Reimer, 1985) Bray, 1995 with 98.4% similarity (12 nucleotide difference, Suppl. material 2).

In addition, two 727-bp sequences were obtained for *P.profundis* sp. nov. from the *cox1* mDNA gene. One polymorphic site was detected between the two sequences. Sequences for the *cox1* mDNA are available only for two genera of Fellodistomidae (*Proctoeces* Odhner, 1911 and *Lintonium* Stunkard & Nigrelli, 1930), which precludes a phylogenetic analysis.

## ﻿Discussion

Members of Fellodistomidae are parasitic in the intestines, pyloric caeca, bile ducts, and gallbladders of marine and occasionally freshwater fishes but also occur as adults in molluscs (bivalves and gastropods) (Bray 2002; Oliva et al. 2018). The family comprises 26 genera with 138 species, of which 39 species are parasites of deep-sea fishes (Klimpel et al. 2009; Glover et al. 2024). *Pseudosteringophorusprofundis* sp. nov., parasitizes the gallbladder of the deep-sea *Macrourusholotrachys*, unlike other genera in the family Fellodistomidae, which typically parasitize the intestines of deep-sea fishes. These genera include *Benthotrema* Manter, 1934, *Pseudobenthotrema* Machida, Kamegai & Kuramochi, 2007, *Hypertrema* Manter, 1960, *Lomasoma* Manter, 1935, *Megenteron* Manter, 1934, *Olssonium* Bray & Gibson, 1980, *Prudhoeus* Bray & Gibson, 1980, *Pseudosteringophorus* Yamaguti, 1940, *Steringophorus* Odhner, 1905, *Steringovermes* Bray, 2004 and *Steringotrema* Odhner, 1911 (Bray 2002; Glover et al. 2024).

The genus *Pseudosteringophorus*, is closely related to the genus *Steringophorus.* The main differences include the vitellaria located immediately lateral to the anterior half of each caecum, between the pharynx level and the ovary to the anterior border at testicular level, and an oval ovary in *Pseudosteringophorus*. In contrast, the vitellaria in *Steringophorus* are located between the level of the ventral sucker and the level just posterior to the testes; in addition, the ovary is multilobulate (Table 2). To date, *Pseudosteringophorus* is monotypic, with *P.hoplognathi* being the type species of the genus. *Pseudosteringophorushoplognathi* was reported parasitizing the intestines of the shallow-water *Oplegnathuspunctatus* and *Oplegnathusfasciatus* (Centrarchiformes: Oplegnathidae) in Japan (Yamaguti 1940; Machida et al. 2007), as well as *Plectorhinchuscinctus* (Temminck & Schlegel, 1843) (Eupercaria*incertae sedis*: Haemulidae) in China (Wang 1982). In addition, *Pseudosteringophorus* sp. has been reported from the intestines of *Ephippusorbis* (Bloch, 1787) (Acanthuriformes: Ephippidae) (Mamaev 1970). Unfortunately, accurate descriptions were not provided by Wang (1982) and Mamaev (1970). Kuramochi (2001) reported an undescribed species of *Pseudosteringophorus* from the deep-sea *Congriscusmegastoma* (Günther, 1877) (Anguilliformes: Congridae). Kuramochi´s specimens differ from *P.profundis* sp. nov. by the size of the ventral sucker as well as the ending of the caecas (see fig. 4 in Kuramochi 2001).

**Table 2.** Taxonomic differences between *Pseudosteringophorus* and *Steringophorus*.

	*Pseudosteringophorus*	*Steringophorus*
Body	fusiform, more pointed at the posterior extremity than at the anterior	large, oval to elongate oval, deep-bodied to dorsoventrally flattened
Oral sucker	terminal, oval with ventral concavity in lateral view	rounded or subglobular and subterminal
Ventral sucker	bowl-shaped, located at middle of body or a little further behind	usually larger than oral sucker, located in anterior half of body
Caeca	narrow, simple, terminating at acetabular-ovarian level	wide to narrow, extent variable, extending to testes, to about middle of post-testicular region or occasionally beyond
Testes	oval, entire, symmetrical, anterior hindbody	oval, entire, indented or deeply lobed, symmetrical to tandem, in anterior or mid-hindbody
Cirrus sac	recurved, claviform, just reaching ventral sucker	oval
Internal seminal vesicle	bipartite	bipartite
Genital atrium	with a diverticulum totally lined with hairs and surrounded by glandular cells	often with a diverticulum
Genital pore	sinistrally submedian, post-bifurcal	anterior margin of ventral sucker, sinistrally submedian
Ovary	rounded or oval, near or overlapping with the right testes or pretesticular, dextrodorsal to ventral sucker	multilobate, just pretesticular
Uterus	post-testicular region	coiled posteriorly to testes
Eggs	tanned, embryonated, eggshells no ornamented	eggshells occasionally ornamented
Vitelline follicles	in form of single field of small follicles between pharynx level and ovary to anterior border testicular level	in two lateral fields between level of ventral sucker and level just posterior to testes

Manter (1954) and Bray (2002) expressed doubts regarding the generic status of *Benthotremahoplognathi* Yamaguti, 1938, which they found to be closely related to *Pseudosteringophorushoplognathi*. Both species are parasites of fishes of the genus *Hoplagnathus* (= *Oplegnathus*). Machida et al. (2007) reviewed Yamaguti’s specimens of *P.hoplognathi* and *B.hoplognathi*, along with their own specimens of *P.hoplognathi*. Their analysis found no significant differences between the two species and suggested that both are synonymous because of the presence of a bipartite internal seminal vesicle, a characteristic in *Pseudosteringophorus.* Meanwhile, Bray (2002) indicated that the genus *Benthotrema* is characterized by a coiled, tubular internal seminal vesicle, and consequently, *B.hoplognathi* should be considered a member of *Pseudosteringophorus*.

Our study provides the first DNA sequences for species of the genus *Pseudosteringophorus* which nest within members of the genus *Steringophorus*, but the position of *S.dorsolineatus* suggests a possible paraphyly, although with low nodal support among *Steringophorus* as previously noted (Pérez-Ponce de León et al. 2018; Bray and Waeschenbach 2020; Cribb et al. 2021). The classification of *S.dorsolineatus*, originally described as *Occultacetabulumdorsolineatum* by Reimer (1985), has been questioned regarding its inclusion in the genus *Steringophorus* (Bray and Waeschenbach 2020; Sokolov et al. 2021). This has led to a proposal to reinstate the genus *Occultacetabulum*, based on differences in ventral sucker morphology, a narrow ventral slit-like opening in contrast to the unspecialized, rounded ventral sucker of *Steringophorus* (Sokolov et al. 2021). Our phylogenetic analyses support the hypothesis of paraphyly.

## ﻿Conclusion

This study provides the first description of a new species of digenean from the family Fellodistomidae from the deep waters of SEPO, infecting the gallbladder of *Macrourusholotrachys*. Our results suggest the need for increasing sampling efforts for other fellodistomid species that are morphologically close to the genus *Pseudosteringophorus*, such as *Benthotrema* and *Pseudobenthotrema*. This would help to clarify and improve the resolution of the *Steringophorus* spp. + *Pseudosteringophorus* clade.

## Supplementary Material

XML Treatment for Pseudosteringophorus profundis

## ﻿Additional information

### Author ORCIDs

Marcelo E. Oliva https://orcid.org/0000-0003-1759-2797

Fabiola A. Sepúlveda https://orcid.org/0000-0002-7876-7231

Rubén Escribano https://orcid.org/0000-0002-9843-7723

Luis A. Ñacari https://orcid.org/0000-0001-9692-8476

### Data availability

All of the data that support the findings of this study are available in the main text or Supplementary Information.

## Supplementary materials

Supplementary material 1 Data on the 28S rDNA sequences used in the phylogenetic analysis Data type xlsx https://binary.pensoft.net/file/1210757This dataset is made available under the Open Database License (http://opendatacommons.org/licenses/odbl/1.0/). The Open Database License (ODbL) is a license agreement intended to allow users to freely share, modify, and use this Dataset while maintaining this same freedom for others, provided that the original source and author(s) are credited.Marcelo E. Oliva, Fabiola A. Sepúlveda, Rubén Escribano, Luis A. Ñacari

Supplementary material 2 Pairwise sequence divergences for 28S rDNA sequences of family Fellostomidae Data type xlsx Explanation note The p-distance is shown as a percentage (below the diagonal) and the raw number of bp-pairwise differences above the diagonal. https://binary.pensoft.net/file/1210758This dataset is made available under the Open Database License (http://opendatacommons.org/licenses/odbl/1.0/). The Open Database License (ODbL) is a license agreement intended to allow users to freely share, modify, and use this Dataset while maintaining this same freedom for others, provided that the original source and author(s) are credited.Marcelo E. Oliva, Fabiola A. Sepúlveda, Rubén Escribano, Luis A. Ñacari

## References

[B1] Akaike H (1973) Maximum likelihood identification of gaussian autoregressive moving average models. Biometrika 60: 255–265. 10.1093/biomet/60.2.255

[B2] Bray RA (2002) Family Fellodistomidae Nicoll, 1909. In: Jones A Bray RA Gibson DI (Eds) , Keys to the Trematoda Vol.1. Wallingford: CABI Publishing and the Natural History Museum, 261–293. 10.1079/9780851995472.0261

[B3] Bray RA (2020) Digenean parasites of deep-sea teleosts: A progress report. International Journal for Parasitology: Parasites and Wildlife 12: 251–264. 10.1016/j.ijppaw.2020.01.007 33101904 PMC7569682

[B4] Bray RA Waeschenbach A (2020) *Steringophorusmerretti* n. sp. (Digenea: Fellodistomidae) from the deep-sea fish *Cataetyxlaticeps* Koefoed (Ophidiiformes: Bythitidae) from the Goban Spur, Northeastern Atlantic Ocean. Systematic Parasitology 97: 321–334. 10.1007/s11230-020-09919-3 32495189 PMC7320048

[B5] Castresana J (2000) Selection of conserved blocks from multiple alignments for their use in phylogenetic analysis. Molecular Biology and Evolution 17: 540–552. 10.1093/oxfordjournals.molbev.a026334 10742046

[B6] Chisholm LA Morgan JAT Adlard RD Whittington ID (2001) Phylogenetic analysis of the Monocotylidae (Monogenea) inferred from 28S rDNA sequences. International Journal for Parasitology 31: 1253–1263. 10.1016/S0020-7519(01)00223-5 11513895

[B7] Cribb TH Martin SB Diaz PE Bray RA Cutmore SC (2021) Eight species of *Lintonium* Stunkard & Nigrelli, 1930 (Digenea: Fellodistomidae) in Australian tetraodontiform fishes. Systematic Parasitology 98: 595–624. 10.1007/s11230-021-10000-w 34536191

[B8] Danovaro R Gambi C Della Croce N (2002) Meiofauna hotspot in the Atacama Trench, eastern South Pacific Ocean. Deep Sea Research Part I: Oceanographic Research Papers 49: 843–857. 10.1016/S0967-0637(01)00084-X

[B9] Danovaro R Company JB Corinaldesi C D’Onghia G Galil B Gambi C Gooday AJ Lampadariou N Luna GM Morigi C Olu K Polymenakou P Ramirez-Llodra E Sabbatini A Sardá F Sibuet M Tselepides A (2010) Deep-sea biodiversity in the Mediterranean Sea: The known, the unknown, and the unknowable. PLoS ONE 5: e11832. 10.1371/journal.pone.0011832 PMC2914020 20689848

[B10] Espínola-Novelo JF Escribano R Oliva ME (2018) Metazoan parasite communities of two deep-sea elasmobranchs: the southern lanternshark, *Etmopterusgranulosus*, and the largenose catshark, *Apristurusnasutus*, in the Southeastern Pacific Ocean. Parasite 25: 53. 10.1051/parasite/2018054 PMC6244290 30457552

[B11] Filatov DA (2002) PROSEQ: A software for preparation and evolutionary analysis of DNA sequence data sets. Molecular Ecology Notes 2: 621–624. 10.1046/j.1471-8286.2002.00313.x

[B12] Fujii T Kilgallen N Rowden A Jamieson A (2013) Deep-sea amphipod community structure across abyssal to hadal depths in the Peru-Chile and Kermadec trenches. Marine Ecology Progress Series 492: 125–138. 10.3354/meps10489

[B13] Gambi C Vanreusel A Danovaro R (2003) Biodiversity of nematode assemblages from deep-sea sediments of the Atacama Slope and Trench (South Pacific Ocean). Deep-Sea Research Part I: Oceanographic Research Papers 50: 103–117. 10.1016/S0967-0637(02)00143-7

[B14] Glover A Higgs N Horton T (2024) World Register of Deep-Sea species (WoRDSS). Fellodistomidae Nicoll, 1909.

[B15] Katoh K Rozewicki J Yamada KD (2019) MAFFT online service: Multiple sequence alignment, interactive sequence choice and visualization. Briefings in Bioinformatics 20: 1160–1166. 10.1093/bib/bbx108 28968734 PMC6781576

[B16] Klimpel S Busch MW Kellermanns E Kleinertz S Palm HW (2009) Metazoan Deep Sea Fish Parasites. Acta Biologica Benrodis.

[B17] Kuramochi T (2001) Digenean Trematodes of Anguilliform and Gadiform Fishes from Deep-Sea Areas of Tosa Bay, Japan. National Science Museum monographs 20: 19–30.

[B18] Leung TLF Donald KM Keeney DB Koehler A V Peoples RC Poulin R (2009) Trematode parasites of Otago Harbour (New Zealand) soft-sediment intertidal ecosystems: Life cycles, ecological roles and DNA barcodes. New Zealand Journal of Marine and Freshwater Research 43: 857–865. 10.1080/00288330909510044

[B19] Machida M Kamegai S Kuramochi T (2007) Fellodistomidae (Trematoda, Digenea) from deep-sea fishes of Japan. Bulletin of the National Science Museum. Series A (Zoology) 33: 93–103.

[B20] Mamaev YL (1970) [Helminths of some commercial fishes in the Gulf of Tong King]. In: Oshmarin PG Mamaev YL Lebedev BI (Eds) Helminths of Animals of South-East Asia.Moscow, Izdatel’stvo Nauka, 127–190. [In Russian]

[B21] Manter H (1954) Some digenetic trematodes from fishes of New Zealand. Transactions of the Royal Society of New Zealand 82: 475–568.

[B22] McLaughlin JP Morton DN Lafferty KD (2020) Parasites in marine food webs. In: Marine Disease Ecology. Oxford University Press, 45–60. 10.1093/oso/9780198821632.003.0002

[B23] Miller SA Dykes DD Polesky HF (1988) A simple salting out procedure for extracting DNA from human nucleated cells. Nucleic Acids Research 16: 1215. 10.1093/nar/16.3.1215 PMC334765 3344216

[B24] Muff S Nilsen EB O’Hara RB Nater CR (2022) Rewriting results sections in the language of evidence. Trends in Ecology and Evolution 37: 203–210. 10.1016/j.tree.2021.10.009 34799145

[B25] Münster J Kochmann J Klimpel S Klapper R Kuhn T (2016) Parasite fauna of Antarctic *Macrouruswhitsoni* (Gadiformes: Macrouridae) in comparison with closely related macrourids. Parasites & Vectors 9: 403. 10.1186/s13071-016-1688-x PMC4955115 27439703

[B26] Ñacari LA Oliva ME (2016) Metazoan parasites of deep-sea fishes from the South Eastern Pacific: Exploring the role of ecology and host phylogeny. Deep-Sea Research Part I: Oceanographic Research Papers 115: 123–130. 10.1016/j.dsr.2016.06.002

[B27] Ñacari LA Escribano R Oliva ME (2022) Endoparasites and diet of the “bigeye grenadier” *Macrourusholotrachys* Günther, 1878 from the deep sea in the Southeastern Pacific Ocean. Deep Sea Research Part I: Oceanographic Research Papers 190: 103903. 10.1016/j.dsr.2022.103903

[B28] Oliva ME Fernández I Oyarzún C Murillo C (2008) Metazoan parasites of the stomach of *Dissostichuseleginoides* Smitt 1898 (Pisces: Nototheniidae) from southern Chile: A tool for stock discrimination? Fisheries Research 91: 119–122. 10.1016/j.fishres.2007.11.012

[B29] Oliva ME Valdivia IM Cárdenas L Muñoz G Escribano R George-Nascimento M (2018) A new species of *Proctoeces* and reinstatement of *Proctoeceshumboldti* George-Nascimento and Quiroga 1983 (Digenea: Fellodistomidae) based on molecular and morphological evidence. Parasitology International 67: 159–169. 10.1016/j.parint.2017.10.004 29079224

[B30] Pérez-Ponce de León G Anglade T Randhawa HS (2018) A new species of *Steringotrema* Odhner, 1911 (Trematoda: Fellodistomidae) from the New Zealand sole *Peltorhamphusnovaezeelandiae* Günther off Kaka point in the Catlins, South Island, New Zealand. Systematic Parasitology 95: 213–222. 10.1007/s11230-018-9773-5 29372441

[B31] Posada D (2008) jModelTest: Phylogenetic Model Averaging. Molecular Biology and Evolution 25: 1253–1256. 10.1093/molbev/msn083 18397919

[B32] Rambaut A Drummond AJ Xie D Baele G Suchard MA (2018) Posterior Summarization in Bayesian Phylogenetics Using Tracer 1.7. Systematic Biology 67: 901–904. 10.1093/sysbio/syy032 29718447 PMC6101584

[B33] Ramírez-Flandes S González CE Aldunate M Poulain J Wincker P Glud RN Escribano R Haond SA Ulloa O (2022) High genetic diversity in the pelagic deep-sea fauna of the Atacama Trench revealed by environmental DNA. bioRxiv: 2022.04.14.488404. 10.1101/2022.04.14.488404

[B34] Ramirez-Llodra E Brandt A Danovaro R De Mol B Escobar E German CR Levin LA Martinez Arbizu P Menot L Buhl-Mortensen P Narayanaswamy BE Smith CR Tittensor DP Tyler PA Vanreusel A Vecchione M (2010) Deep, diverse and definitely different: Unique attributes of the world’s largest ecosystem. Biogeosciences 7: 2851–2899. 10.5194/bg-7-2851-2010

[B35] Reimer L (1985) *Occultacetabulumdorsolineatum* ng, n. sp. (Occultacetabulinae n. subf.), a fellodistomine digenean from a deep-sea fish in the Mozambique Channel (in German). Angewandte Parasitologie 26: 107–109.

[B36] Rodriguez L George-Nascimento M (1996) La fauna de parásitos metazoos del bacalao de profundidad *Dissostichuseleginoides* Smitt, 1898 (Pisces: Nototheniidae) en Chile central: aspectos taxonómicos, ecológicos y zoogeográficos. Revista Chilena de Historia Natural 69: 21–33.

[B37] Ronquist F Teslenko M Van Der Mark P Ayres DL Darling A Höhna S Larget B Liu L Suchard MA Huelsenbeck JP (2012) MrBayes 3.2: Efficient Bayesian Phylogenetic Inference and Model Choice Across a Large Model Space. Systematic Biology 61: 539–542. 10.1093/sysbio/sys029 22357727 PMC3329765

[B38] Sabbatini A Morigi C Negri A Gooday AJ (2002) Soft-shelled benthic foraminifera from a hadal site (7800 m water depth) in the Atacama Trench (SE Pacific): preliminary observations. Journal of Micropalaeontology 21: 131–135. 10.1144/jm.21.2.131

[B39] Salinas X González MT Acuña E (2008) Metazoan parasites of the thumb grenadier *Nezumiapulchella*, from the south-eastern Pacific, off Chile, and their use for discrimination of host populations. Journal of Fish Biology 73: 683–691. 10.1111/j.1095-8649.2008.01967.x

[B40] Sokolov SG Shchenkov S V. Gordeev II (2021) A phylogenetic assessment of *Pronoprymna* spp. (Digenea: Faustulidae) and Pacific and Antarctic representatives of the genus *Steringophorus* Odhner, 1905 (Digenea: Fellodistomidae), with description of a new species. Journal of Natural History 55: 867–887. 10.1080/00222933.2021.1923852

[B41] Tamura K Stecher G Peterson D Filipski A Kumar S (2013) MEGA6: Molecular evolutionary genetics analysis version 6.0. Molecular Biology and Evolution 30: 2725–2729. 10.1093/molbev/mst197 24132122 PMC3840312

[B42] Wang PQ (1982) Some digenetic trematodes of marine fishes from Fujian Province, China. Oceanologia et Limnologia Sinica 13: 179–194. [In Chinese]

[B43] Weston JNJ Espinosa-Leal L Wainwright JA Stewart ECD González CE Linley TD Reid WDK Hidalgo P Oliva ME Ulloa O Wenzhöfer F Glud RN Escribano R Jamieson AJ (2021) *Eurythenesatacamensis* sp. nov. (Crustacea: Amphipoda) exhibits ontogenetic vertical stratification across abyssal and hadal depths in the Atacama Trench, eastern South Pacific Ocean. Marine Biodiversity 51: 51. 10.1007/s12526-021-01182-z PMC8120496 34007343

[B44] Yamaguti S (1938) Studies on the helminth fauna of Japan. Part 21. Trematodes of fishes, IV. Published by author, Japan, 139 pp.

[B45] Yamaguti S (1940) Studies on the helminth fauna of Japan. Part 31. Trematodes of fishes, VII. Journal of Zoology 9: 35–108.

